# Predictors for mechanical ventilation and short-term prognosis in patients with Guillain-Barré syndrome

**DOI:** 10.1186/s13054-015-1037-z

**Published:** 2015-09-02

**Authors:** Xiujuan Wu, Chunrong Li, Bing Zhang, Donghui Shen, Ting Li, Kangding Liu, Hong-Liang Zhang

**Affiliations:** Neuroscience Center, Department of Neurology, the First Hospital of Jilin University, Jilin University, Xinmin Street 71#, 130021 Changchun, China; Department of Neurobiology, Care Sciences and Society, Karolinska Institute, Stockholm, Sweden

## Abstract

**Introduction:**

Guillain-Barré syndrome (GBS) is an immune-mediated disorder of the peripheral nervous system. Respiratory failure requiring mechanical ventilation (MV) is a serious complication of GBS. Identification of modifiable risk factors for MV and poor short-term prognosis in mechanically ventilated patients with GBS may contribute to the individualized management and may help improve the outcome of the patients.

**Methods:**

We retrospectively analyzed the clinical data of 541 patients who were diagnosed with GBS from 2003 to 2014. Independent predictors for MV and short-term prognosis in mechanically ventilated patients were identified via multivariate logistic regression analysis.

**Results:**

The mean age was 41.6 years with a male predilection (61.2 %). Eighty patients (14.8 %) required MV. Multivariate analysis revealed that shorter interval from onset to admission (*p* < 0.05), facial nerve palsy (*p* < 0.01), glossopharyngeal and vagal nerve deficits (*p* < 0.01) and lower Medical Research Council (MRC) sum score at nadir (*p* < 0.01) were risk factors for MV; disease occurrence in summer (*p* < 0.01) was a protective factor. As to prognostic factors, absence of antecedent infections (*p* < 0.01) and lower MRC sum score at nadir (*p* < 0.01) were predictors of poor short-term prognosis in mechanically ventilated patients regardless of treatment modality. We further investigated the predictors of poor short-term prognosis in patients requiring MV with different nadir MRC sum scores. Combined use of intravenous corticosteroids with intravenous immunoglobulin (odds ratio 10.200, 95 % confidence interval 1.068–97.407, *p* < 0.05) was an independent predictor of poor short-term prognosis in mechanically ventilated patients with a nadir MRC sum score from 0 to 12 points, regardless of existence of antecedent infection.

**Conclusions:**

Clinical predictors of MV and poor short-term prognosis in mechanically ventilated GBS patients were distinct. Add-on use of intravenous corticosteroids was a risk factor for poor short-term prognosis in mechanically ventilated patients with a nadir MRC sum score from 0 to 12 points.

## Introduction

Guillain-Barré syndrome (GBS) is triggered by infectious or noninfectious agents and is usually considered to be an immune-mediated disorder of the peripheral nervous system [1, 2]. As one of the common causes of acute neuromuscular paralysis, GBS represents a clinical syndrome and most patients with GBS present with progressively flaccid, symmetric and ascending weakness of extremities. Respiratory failure requiring mechanical ventilation (MV) is a common and serious short-term complication of GBS with a reported incidence ranging from 20 to 30 % [3–5]. Early prediction of MV in patients with GBS is of great importance to enable clinicians to tailor supportive care and individualized treatment. Increasing studies have focused on the clinical predictors of MV in patients with GBS. Multiple clinical parameters have been found to serve as predictors of MV from different studies, including time from onset to admission of <7 days, inability to cough and stand, inability to lift the elbows or head from the bed, cranial nerve deficits, increased liver enzyme levels, anti-GQ1b antibodies, low vital capacity, phrenic nerve compound muscular amplitude potential (CMAP) latency, and the proximal/distal compound muscular amplitude potential (p/d CMAP) ratio of the peroneal nerve [5–11]. Severe GBS, including that requiring MV, is usually associated with unfavorable residual sequelae or mortality, and early identification of modifiable risk factors for the poor prognosis may help decrease the incidence of residual sequelae and mortality. Risk factors for mortality in mechanically ventilated patients with GBS include older age, autonomic dysfunction and pulmonary complications [12]. In addition, hypokalemia and bleeding were also reported to be associated with increased incidence of mortality in GBS patients requiring MV [13]. A previous study demonstrated a seasonal variation in the recovery of patients with GBS requiring MV [14]. Considering the pivotal role of early identification of modifiable risk factors for MV and poor prognosis, we herein investigated the clinical predictors of poor short-term prognosis in mechanical ventilated patients with GBS, as well as the risk factors for MV.

## Methods

### Study design and setting

This retrospective study was approved by the ethics committee of the First Hospital of Jilin University, Changchun, China. A written informed consent was acquired from all the patients. The records of the patients were anonymized and de-identified prior to analysis. From 2003 to 2014, patients who were admitted to the Department of Neurology of the First Hospital of Jilin University and fulfilled the diagnostic criteria of GBS were enrolled [15]. Patients were excluded if they met one of the following exclusion criteria: younger than 16 years; diagnosed as either Miller Fisher syndrome, chronic inflammatory demyelinating polyradiculoneuropathy, Bickerstaff encephalitis, or critical illness polyneuropathy/myopathy (CIP/CIM) [16]. In addition, patients with porphyric neuropathy were excluded as this neuropathic condition typically occurs in association with other features of an acute attack of hepatic porphyria and can be differentiated from GBS by clinical features, such as a proximal predilection of asymmetric weakness, accompanying psychiatric abnormalities, and laboratory examinations (urine porphyrins) [17]. Patients who were discharged within 3 days were ruled out because they might not reach the nadir when discharged. Missing data due to the short hospital stay is another reason for this exclusion. For all the included subjects, data about age, sex, preceding infections (mainly including upper respiratory infection and diarrhea), time from onset to admission and time from onset to nadir, clinical severity assessed by the Hughes Functional Grading Scale (HFGS), muscle weakness evaluated by the Medical Research Council (MRC) sum score, sensory disturbances and reflexes in arms and legs, cranial nerve deficits, autonomic dysfunctions (fluctuating blood pressure, tachyarrhythmia and bradyarrhythmia, and abnormal sweating) and pain, as well as treatment modality and complications during hospitalization were collected. Intravenous immunoglobulin (IVIg) is the first-line treatment option for GBS patients who were unable to walk independently (HFGS ≥3) in our department, while intravenous corticosteroids as an add-on therapy were administrated to some of the severe patients as determined by the neurologists. Of note, if the severe patients refused IVIg, they were prescribed either intravenous corticosteroids or supportive treatments.

### Evaluation of clinical severity and functional impairment

The clinical severity and functional impairment were evaluated for all the enrolled subjects. The HFGS score was used to assess functional disability, which was defined as follows [18]: 0, healthy state; 1, minor symptoms and capable of running; 2, able to walk 5 m or more without assistance but unable to run; 3, able to walk 5 m across an open space with help; 4, bedridden or chair-bound; 5, requiring assisted ventilation for at least part of the day; 6, dead. Muscle weakness was evaluated by the MRC sum score of six bilateral muscles in arms and legs, ranging from 0 (tetraparalytic) to 60 (normal strength) [19]. Nadir of disease was defined as the highest HFGS score or the lowest MRC sum score.

### Grouping and short-term prognosis assessment

Enrolled patients were divided into two groups according to the requirement or not for MV, i.e., GBS patients requiring MV (Group MV) and patients not requiring MV (Group NV). In Group MV, the patients could be further divided into two subgroups according to their severity 4 weeks after the treatment, i.e., patients with a good short-term prognosis (Subgroup 1) and patients with a poor short-term prognosis (Subgroup 2). The ability to walk with assistance at discharge (HFGS ≤3) was considered to be a good short-term prognosis.

### Statistical analysis

Statistical analysis was performed with SPSS version 17.0 software (SPSS, IBM, West Grove, PA, USA). Categorical data are presented as proportions, while continuous data are presented as means and standard deviations or means and interquartile ranges depending on the distribution of the data. Differences in proportions were tested by the Chi-square tests and differences in continuous variables were tested by student *t*-tests. Independent predictors of MV and short-term prognosis in mechanically ventilated patients with GBS were determined by using multivariate logistic regression analysis. The final models for independent predictors were the variables with statistically significant contributions obtained from univariate analysis. For all statistical tests, *p* < 0.05 was considered to be significant.

## Results

### Demographic features of enrolled patients with GBS

**Fig. 1:**
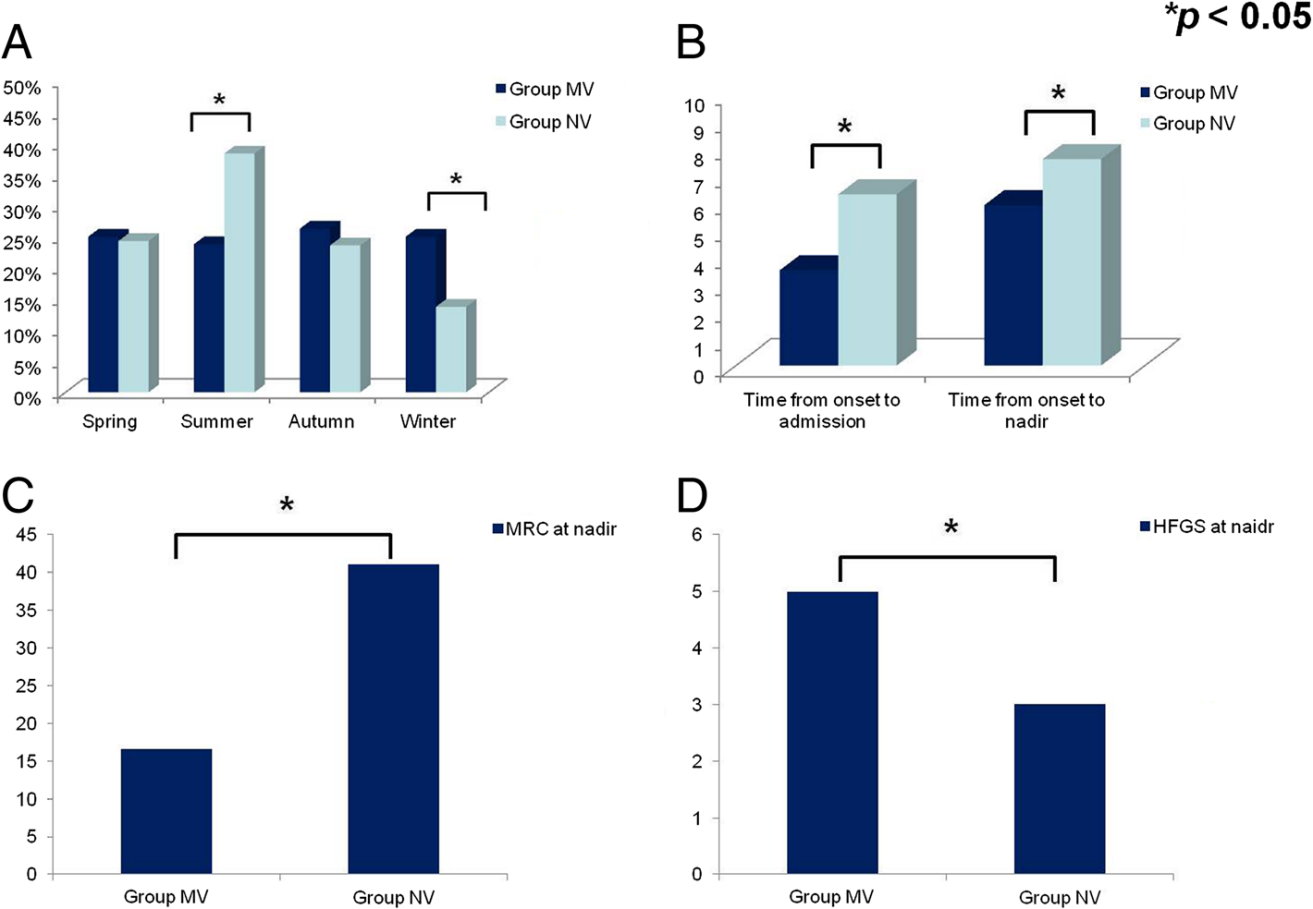
Comparisons between patients with and without mechanical ventilation. **a** The seasonal distribution in GBS occurrence was different between Group MV and Group NV. More GBS patients with mechanical ventilation were found in winter, while those without mechanical ventilation were more common in summer (both *p* < 0.05). **b** Time from onset to admission and time from onset to nadir were both shorter in Group MV (3.5 versus 6.3 and 5.9 versus 7.6; both *p* < 0.05). As assessed by **c** the MRC sum score and **d** HFGS, more severe disease severity was found in Group MV (MRC, 16.6 versus 41.0; HFGS, 5 versus 3.0; both *p* < 0.05). *Group MV* GBS patients with mechanical ventilation, *Group NV* GBS patients did not require mechanical ventilation, *HFGS* Hughes Functional Grading Scale, *MRC* Medical Research Council (sum score)

A total of 541 adult patients were enrolled. The mean age was 41.6 years with a male preponderance (61.2 %). Eighty (14.8 %) patients requiring MV during hospitalization were placed into Group MV, while the remaining 461 patients were in the Group NV. Comparisons between Group MV and Group NV are demonstrated in Table 1. We found that cranial nerve involvement was more common in Group MV (66.3 % versus 39.9 %, *p* < 0.05), as was a longer hospitalization period (38.7 versus 14.6, *p* < 0.05). Seasonal distribution was also significantly different between the two groups, as shown in Fig. 1a. More patients with MV (Group MV) were admitted in winter while more patients without MV (Group NV) were admitted in summer (both *p* < 0.05). Time from onset to admission and time from onset to nadir was both shorter in the Group MV (3.5 versus 6.3 and 5.9 versus 7.6; both *p* < 0.05), as shown in Fig. 1b. In addition, muscle weakness assessed by MRC sum score and disability evaluated by HFGS indicated more severe disease severity in mechanically ventilated patients (Group MV) (MRC, 16.6 versus 41.0, *p* < 0.05; HFGS, 5 versus 3.0, *p* < 0.05; Fig. 1c and d).

**Table 1:** Comparisons between GBS patients with and without mechanical ventilation

Variable	Group MV (n = 80)	Group NV (n = 461)	*p* value
Age (years)	43.8 ± 16.7	41.2 ± 15.3	>0.05
Male	51 (63.8 %)	280 (60.7 %)	>0.05
Antecedent infections	55 (68.75 %)	301 (65.3 %)	>0.05
Cranial nerve involvement	53 (66.25 %)	184 (39.91 %)	<0.05
Facial nerve	43 (81.1 %)	126 (68.5 %)	<0.05
Glossopharyngeal and vagus nerves	30 (56.6 %)	50 (27.2 %)	<0.05
Oculomotor and/or abducent nerve	12 (15 %)	57 (12.4 %)	>0.05
Sensory disturbance	36 (45 %)	249 (54.0 %)	>0.05
Autonomic dysfunction	7 (8.8 %)	20 (4.3 %)	>0.05
Pain	7 (8.8 %)	45 (9.8 %)	>0.05
Hyporeflexia or areflexia	79 (98.8 %)	434 (94.1 %)	>0.05
Hospital stay (days)	38.7 ± 28.3	14.6 ± 6.3	<0.05
Treatment modality			>0.05
IVIg	39 (48.8 %)	216 (46.9 %)	
IVIg + intravenous corticosteroids	29 (36.2 %)	72 (15.6 %)	
Intravenous corticosteroids	6 (7.5 %)	90 (19.5 %)	
Supportive treatment	6 (7.5 %)	83 (18.0 %)	

*GBS* Guillain-Barré syndrome, *Group MV* GBS patients with mechanical ventilation, *Group NV* GBS patients did not require mechanical ventilation, *IVIg* intravenous immunoglobulin

### Clinical predictors of MV

Independent predictors of MV in patients with GBS were determined by multivariate logistic regression analysis. The univariate analysis revealed that the following clinical parameters including seasons (summer and winter), time from onset to admission, time from onset to nadir, presence of cranial nerve involvement (facial, glossopharyngeal and vagal nerve deficits), MRC sum score at nadir and treatment modality (IVIg plus intravenous corticosteroids) were statistically significant (*p* < 0.05). Further, variables including time from onset to admission, disease occurrence in summer, presence of cranial nerve involvement (facial, glossopharyngeal and vagus nerve deficits), and MRC sum score at nadir were identified as significant by the multivariate analysis. As shown in Table 2, we found that shorter time from onset to admission, presence of facial, glossopharyngeal and vagal nerve deficits, and lower MRC sum score at nadir were risk factors for MV in patients with GBS, while disease occurrence in summer was a protective factor.

**Table 2:** Independent predictors for mechanical ventilation in patients with GBS

Variable	Regression coefficient (95 % CI)	*p* value	Exp (B)
Time from onset to admission	−0.117 (0.808–0.980)	<0.05	0.899
Disease occurrence in summer	−1.520 (0.102–0.469)	<0.01	0.219
Facial nerve involvement	0.921 (1.281–4.926)	<0.01	2.512
Glossopharyngeal and vagus nerves involvement	1.703 (2.561–11.773)	<0.01	5.491
MRC at nadir	−0.099 (0.886–0.926)	<0.01	0.906

*CI* confidence interval, *GBS* Guillain-Barré syndrome, *MRC* Medical Research Council (sum score)

### Clinical predictors of poor short-term prognosis in mechanically ventilated patients

**Fig. 2:**
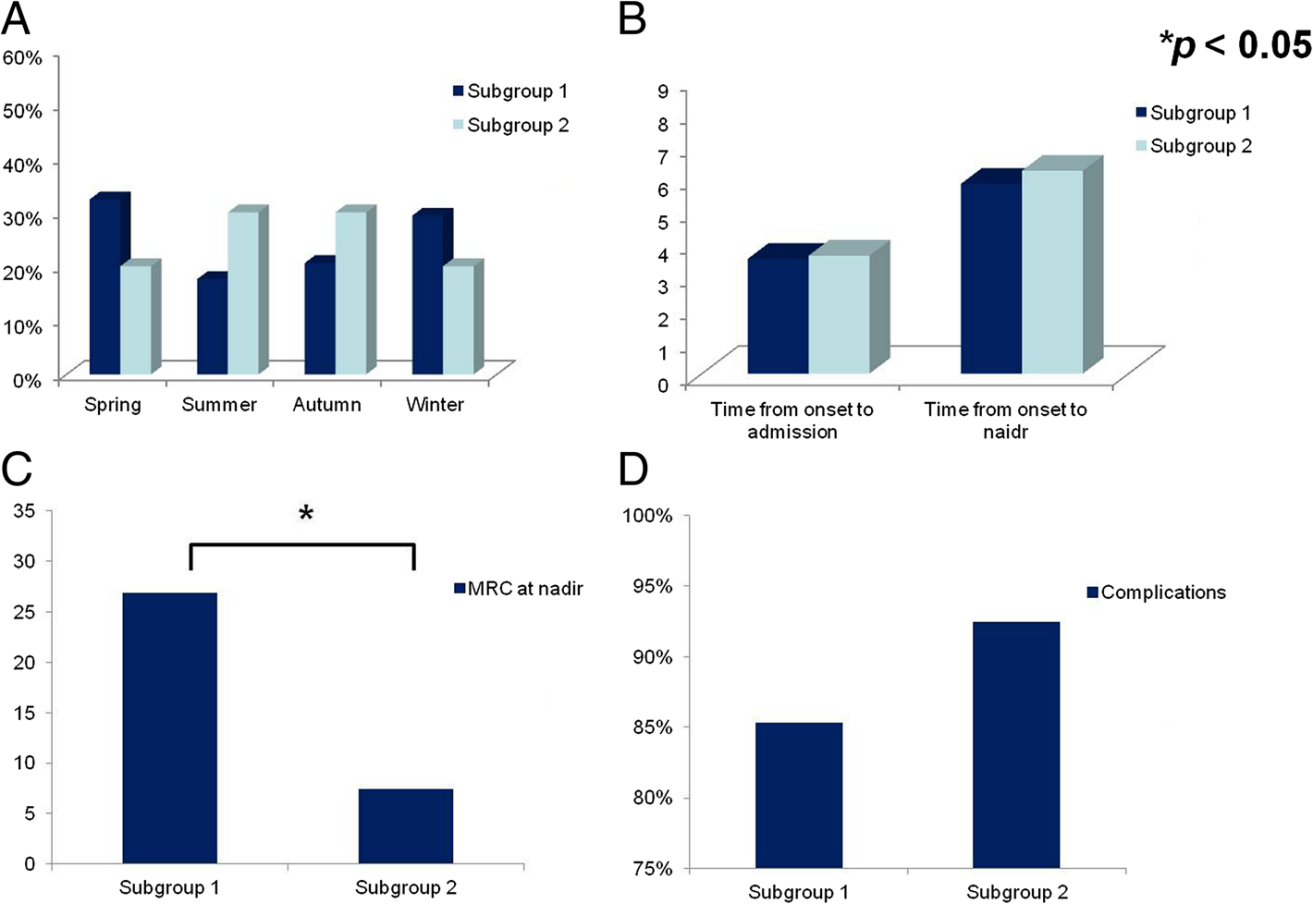
Comparisons of mechanically ventilated patients with good and poor short-term prognoses. **a** Seasonal distribution in the mechanically ventilated GBS patients with good short-term prognosis (Subgroup 1) was similar to that in the poor-short-term prognosis (Subgroup 2) (spring, 32.4 % versus 20 %; summer, 17.6 % versus 30 %; autumn, 20.6 % versus 30 %; winter, 29.4 % versus 20 %; all *p* > 0.05). **b** Time from onset to admission (3.5 versus 3.6, *p* > 0.05) and time from onset to nadir (5.8 versus 6.2, *p* > 0.05) were not significantly different. **c** MRC at nadir was significantly lower in the poor short-term prognosis group compared with the good short-term prognosis group (26.9 versus 7.4, *p* < 0.05). **d** As the infection complications during hospitalization were related to the prognosis of patients, we further compared the incidence of complications and found it was similar between two groups (85.3 % versus 92.5 %, *p* > 0.05). *MRC* Medical Research Council (sum score)

Patients who were able to walk with assistance at discharge (HFGS ≤3) were considered to have a good short-term prognosis. Patients in Group MV were further divided into two subgroups according to their short-term prognosis at discharge, i.e., patients with a good short-term prognosis (Subgroup 1) and patients with poor short-term prognosis (Subgroup 2). As our aim was to identify the clinical predictors of poor short-term prognosis in mechanically ventilated patients and the treatment that might be associated with the short-term prognosis, we excluded six patients who were discharged within 3 days in Group MV. Among the remaining 74 patients, 33 patients received IVIg treatment, 29 patients were treated with IVIg combined with intravenous corticosteroids, 6 patients received intravenous corticosteroids and 6 patients received supportive treatment instead of immunotherapy. Comparisons between the Subgroup 1 and Subgroup 2 are demonstrated in Table 3. Glossopharyngeal and vagal nerve deficits were higher in the good short-term prognosis group (52.9 % versus 30 %, *p* < 0.05). Seasonal distribution and the time from onset to admission and to nadir were not different (*p* > 0.05) (Fig. 2a and b). Of note, the MRC sum score at nadir was significantly lower in the poor short-term prognosis group compared with the good short-term prognosis group (7.4 versus 26.9, *p* < 0.05; Fig. 2c). As complications during hospitalization might affect the prognosis of patients with GBS, we compared the incidence of complications and found that complications occur comparably between the two groups (85.3 % versus 92.5 %, *p* > 0.05; Fig. 2d).

**Table 3:** Comparisons between mechanically ventilated GBS patients with good and poor short-term prognoses

Variable	Subgroup 1 (*n* = 34)	Subgroup 2 (*n* = 40)	*p* value
Age (years)	43.9 ± 16.3	41.9 ± 17.3	>0.05
Male	25 (73.5 %)	23 (57.5 %)	>0.05
Antecedent infections	28 (82.4 %)	21 (52.5 %)	>0.05
Cranial nerve involvement	26 (76.5 %)	24 (60 %)	>0.05
Facial nerve	18 (52.9 %)	22 (55 %)	>0.05
Glossopharyngeal and vagus nerves	18 (52.9 %)	12 (30 %)	<0.05
Sensory disturbance	16 (47.1 %)	17 (42.5 %)	>0.05
Autonomic dysfunction	3 (8.8 %)	2 (5 %)	>0.05
Pain	3 (8.8 %)	4 (10 %)	>0.05
Hyporeflexia or areflexia	33 (97.1 %)	39 (97.5 %)	>0.05
Hospital stay (days)	31.0 ± 16.1	51.7 ± 31.2	<0.05
Treatment modality			>0.05
IVIg	18 (52.9 %)	15 (37.5 %)	
IVIg + intravenous corticosteroids	11 (32.4 %)	18 (45 %)	
Intravenous corticosteroids	2 (5.9 %)	4 (10 %)	
Supportive treatment	3 (8.8 %)	3 (7.5 %)	

*GBS* Guillain-Barré syndrome, *IVIg* intravenous immunoglobulin, *Subgroup 1* mechanically ventilated GBS patients with good short-term prognosis, *Subgroup 2* mechanically ventilated GBS patients with poor short-term prognosis

Univariate analysis revealed that absence of antecedent infections, presence of glossopharyngeal and vagal nerve deficits, and the MRC sum score at nadir were statistically significant variables in predicting poor short-term prognosis in MV patients (*p* < 0.05). Furthermore, absence of antecedent infections and the nadir MRC sum score were also identified as poor prognostic characteristics by multivariate analysis (*p* < 0.05) (Table 4).

**Table 4:** Independent predictors for poor short-term prognosis in mechanically ventilated GBS patients

Variable	Regression coefficient (95 % CI)	*p* value	Exp (B)
Antecedent infections	−1.555 (0.050–0.892)	<0.01	0.211
MRC at nadir	−0.109 (0.852–0.943)	<0.01	0.896

*CI* confidence interval, *GBS* Guillain-Barré syndrome, *MRC* Medical Research Council (sum score)

### Add-on use of intravenous corticosteroids was an independent predictor of poor short-term prognosis in severely paralyzed patients requiring MV

**Fig. 3:**
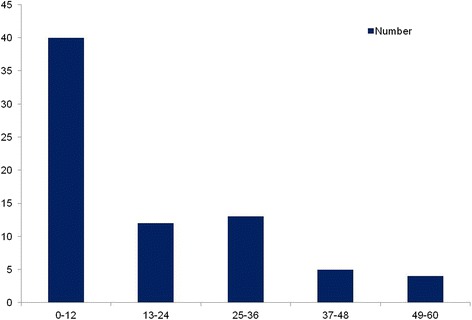
Distribution of mechanically ventilated patients with different nadir MRC sum scores. There were 40 patients with a nadir MRC sum score from 0 to 12 points, corresponding to muscle strength less than 1/5 grade. In addition, 12 patients with a nadir MRC sum score from 13 to 24 points, 13 with a nadir MRC sum score from 25 to 36 points, 5 with a nadir MRC sum score from 37 to 48 points, and 4 with a nadir MRC sum score from 49 to 60 points

We evaluated the effect of added steroid therapy to the outcome in patients with severe muscle weakness at nadir (40 patients, MRC sum score 0 to 12 points) (Fig. 3). These treatments consisted of IVIg therapy (16 cases), IVIg combined with intravenous corticosteroids (18 cases), intravenous corticosteroids alone (2 cases), or supportive treatment alone (4 cases). Only the combination therapy was an independent predictor of poor short-term prognosis regardless of the existence of antecedent infections in these patients (odds ratio 10.200, 95 % confidence interval 1.068–97.407, *p* < 0.05). Thus add-on use of intravenous corticosteroids predicted poor short-term prognosis in severely paralyzed patients requiring MV.

## Discussion

In this study, we searched for predictors for MV and short-term prognosis in mechanically ventilated patients with GBS. Risk factors for MV included shorter interval from onset to admission, presence of facial, glossopharyngeal and vagal nerve deficits, and a lower MRC sum score at nadir; disease occurrence in summer was a protective factor. For prognostic factors in patients requiring MV, we found that absence of antecedent infections and a lower MRC sum score at nadir served as predictors of poor short-term prognosis regardless of treatment options. Considering the impact of nadir MRC sum score on prognosis, we further investigated the predictors for short-term prognosis in mechanically ventilated patients with different nadir MRC sum score ranges and found that add-on use of intravenous corticosteroids was an independent predictor for poor short-term prognosis of patients with a MRC sum score from 0 to 12 points, regardless of the existence of antecedent infection in mechanically ventilated patients.

GBS is a potentially self-limiting disorder and most patients either recover completely or retain only minor residual symptoms. However, respiratory failure requiring MV is a serious short-term complication of GBS with an incidence of 20 to 30 % [3–5]. The incidence of MV in our study was 14.8 %, which was lower than the above-mentioned. This difference might be due to the distribution of different subtypes of GBS. Acute inflammatory demyelinating polyneuropathy (AIDP) is common in many European countries while acute motor axonal neuropathy (AMAN) is particularly prevalent in East Asia [20]. In particular, AIDP and AMAN are distinct from each other in terms of the immunopathogenesis, electrophysiological findings, pathological changes and clinical features; e.g., patients with AMAN less frequently have cranial nerve involvement, and presence of facial and/or bulbar weakness are predictors for MV in patients with GBS [20, 21]. In this regard, the lower incidence of MV in our study might be caused by the distribution of the different subtypes of GBS in different countries. Another reason for the disparity regarding the incidence of MV is likely the different ratio of mildly affected GBS patients (able to walk throughout the disease course) that are diagnosed in different countries and centers. The Department of Neurology of the First Hospital of Jilin University is the largest center for the diagnosis and treatment of GBS in northeast China. IVIg treatment is usually initiated at a very early stage immediately a clinical diagnosis is established, which may hinder the progression of disease course.

Early prediction of MV is important, in that 60 % of the patients with MV developed major complications, including pneumonia, sepsis, gastrointestinal bleeding, and so forth; early identification and intervention may reduce the risk of complications and improve the outcome of patients [22–25]. Accumulating studies have investigated the predictors of MV in patients with GBS. We herein revealed that shorter interval from onset to admission, facial nerve palsy, glossopharyngeal and vagus nerve deficits, and lower MRC sum score at nadir were risk factors for MV, which was consistent with previous findings [5, 10, 11, 21]. Recently, Kannan Kanikannan and colleagues have developed an NSB score model including neck weakness, single breath count, and bulbar palsy to accurately predict the requirement of MV [11]. In addition, we first identified that disease occurrence in summer was a protective factor for MV with unclear mechanisms. This finding warrants further investigation.

Despite the fact that IVIg and plasma exchanges are effective options, patients with GBS still have non-negligible neurological sequelae. Thus, early identification of modifiable risk factors for prognosis in mechanically ventilated patients may improve the outcome of these patients. There have been few studies investigating the prognostic factors of GBS patients who require MV. Predictors for mortality in mechanically ventilated patients with GBS include older age, autonomic dysfunction and pulmonary complications, while a younger age is correlated with a good outcome in these patients [12]. Hypokalemia and bleeding were also reported to be associated with increased incidence of mortality in GBS patients requiring MV [13]. A seasonal variation has been reported in the recovery of patients with GBS requiring MV [14]. Patients admitted in spring usually had the fastest recovery while those in winter had the slowest recovery [14]. In our study, glossopharyngeal and vagus nerve deficits were found in 80 (14.8 %) patients and served as a predictor of MV which was consistent with a previous study [21]. It is reasonable to speculate that glossopharyngeal and vagus nerve deficits would be associated with a poor prognosis in patients with GBS. Of note, the prognostic factors for GBS patients with and without MV might be different. We found that more patients with glossopharyngeal and vagal nerve deficits in the good short-term prognosis subgroup (*p* = 0.045), and the univariate analysis also revealed that glossopharyngeal and vagal nerve deficits were statistically significant. However, this was not validated by the multivariate analysis, implying that glossopharyngeal and vagal nerve deficits did not serve as a predictor for poor short-term prognosis in GBS patients who need MV. The prognostic impact of glossopharyngeal and vagal nerve deficits on GBS patients requiring MV still needs further investigation. In addition, absence of antecedent infections (i.e., GBS is triggered by noninfectious agents, such as intravenous ganglioside) was found to serve as a predictor for poor short-term prognosis in mechanically ventilated patients with GBS. This finding is consistant with our previous study which demonstrated more severe manifestations and poorer short-term prognosis of ganglioside-associated GBS [26], and is in accordance with the study by Lin et al. [27]. They found that the presence of prodromal upper respiratory tract infection was correlated with a better outcome; however, their enrolled subjects were not limited to mechanically ventilated patients [27].

Although IVIg is proven effective treatment, GBS patients could have severe clinical signs or residual functional deficits. Corticosteroids as immunosuppressant and anti-inflammatory agents have been recommended in treatment of severe or protracted GBS cases [28]. In our study, most of the GBS patients requiring MV received either IVIg or combination therapy. Previously, we found that IVIg treatment was superior to combined use of IVIg with intravenous corticosteroids in treating mechanically ventilated patients with GBS (unpublished data). In this study, we found a lower MRC sum score at nadir predicted poor short-term prognosis in GBS patients requiring MV regardless of treatment options. Considering the impact of nadir MRC sum score on prognosis and that combination therapy might be detrimental to specific GBS populations, we further investigated the prognostic factor in mechanically ventilated patients with different nadir MRC sum score ranges. We found that only the combination of IVIg with intravenous corticosteroids was an independent predictor of poor short-term prognosis in those patients with a nadir MRC sum score from 0 to 12 points, i.e., who were severely paralytic. The detrimental effects of corticosteroids might be due to their effect on denervated muscle, or the inhibition of macrophage repair processes, or hyperglycemia caused by corticosteroids [29–32]. In this regard, add-on use of corticosteroids is not suitable for mechanically ventilated patients who are severely paralytic. However, due to the limited sample size, predictors of short-term prognosis in mechanically ventilated GBS patients with different nadir MRC sum scores require further investigation.

There are limitations to our study. First, although the manifestations of CIP/CIM usually resemble those of GBS, the albumino-cytologic dissociation in the cerebrospinal fluid and serum creatine kinase level contribute to the differential diagnosis between GBS and CIP/CIM. However, the development of CIP/CIM during hospitalization in severe GBS patients is difficult to identify, which might adversely affect the prognosis. Second, as a retrospective study, some clinical parameters which have been reported to be predictors of MV were unavailable in our cohort, such as anti-GQ1b antibodies, vital capacity, phrenic nerve CMAP latency, and the p/d CMAP ratio of the peroneal nerve [6, 9, 11]. Third, the sample size for stratified analysis is too small to reach a conclusion with strong statistic significance in our study. In addition, as axonal lesion patterns have been demonstrated as one of the poor prognostic factors for patients with GBS [33], the electrophysiological data might also serve as predictors for poor short-term prognosis in GBS patients requiring MV. Similarly, duration of MV might be an important clinical parameter in predicting the prognosis in patients requiring MV. However, due to the retrospective nature of our study, we failed to make a follow-up on patients who still needed MV when they were discharged and those who had not received electrophysiological examination. Thus, the duration of MV and the electrophysiological data were unavailable in some patients. The prognostic value of duration of MV and subtypes of GBS awaits further validation.

## Conclusions

Clinical predictors for MV and poor short-term prognosis in mechanically ventilated patients with GBS were distinct. Add-on use of intravenous corticosteroids was an independent risk factor for poor short-term prognosis in mechanically ventilated patients with a nadir MRC sum score from 0 to 12 points.

## Key messages

- The short interval from onset to admission, presence of facial nerve palsy, glossopharyngeal and vagal nerve deficits and lower nadir Medical Research Council (MRC) sum score are risk factors, while disease occurrence in summer is a protective factor for mechanical ventilation (MV) in patients with Guillain-Barré syndrome (GBS).
- As to prognostic factors, absence of antecedent infections and lower MRC sum score at nadir are predictors for poor short-term prognosis in mechanically ventilated patients with GBS.
- Add-on use of intravenous corticosteroids is an independent predictor of poor short-term prognosis in mechanically ventilated GBS patients with a nadir MRC sum score from 0 to 12 points, i.e., who were severely paralytic.

## Abbreviations

AIDP: Acute inflammatory demyelinating polyneuropathy
AMAN: Acute motor axonal neuropathy
CIM: Critical illness myopathy
CIP: Critical illness polyneuropathy
CMAP: Compound muscular amplitude potential
GBS: Guillain-Barré syndrome
HFGS: Hughes Functional Grading Scale
IVIg: Intravenous immunoglobulin
MRC: Medical Research Council
MV: Mechanical ventilation
p/d: Proximal/distal
